# Datasets evidencing research on classroom practice in L2 disciplinary writing

**DOI:** 10.1016/j.dib.2019.104222

**Published:** 2019-07-09

**Authors:** Godwin Ioratim-Uba

**Affiliations:** School of Education, University of Nottingham, UNNC, Ningbo, China

**Keywords:** Classroom intervention, Pedagogy, Data, Disciplinary writing

## Abstract

L2 writing in the disciplines at the university level can be impacted through classroom intervention which uses mixed pedagogical paradigms. In connection to such intervention, this data article presents two datasets, from a classroom practice, which evidence disciplinary writing acquisition. The first dataset consists of pre-and post-classroom intervention abstracts, written by L2 PhD students from different disciplines at a Sino-British University. The second dataset is made up of qualitative questionnaire responses from the students about their experiences of the classroom intervention. The datasets can be used to understand the nature of discourse-structural and linguistic feature changes which take place after pedagogical intervention involving mature learners from mixed disciplines. The data can also provide insight into the impact of classroom teaching approaches on the construction and communication of knowledge by novice writers in their disciplines.

**Table:** Specifications table

Subject area	*Education*
More specific subject area	*Disciplinary witting pedagogy*
Type of data	*Tables and figures*
How data was acquired	*Students' writing activities before, during and after classroom teaching and learning; questionnaire survey; assessed written work; paired t test analysis of scores from assessment; and AntConc (Windows 3.5.7) software application for corpus analysis.*
Data format	*Raw and analyzed.*
Experimental factors	*Triple-blind marking of anonymized written abstract; double-checked thematic coding of questionnaire qualitative data*
Experimental features	*Paired t test employed to determine if or not the mean changes across time, from the pre- to the post-intervention written abstracts, differ significantly.*
Data source location	*A Sino-British university in Ningbo, China.*
Data accessibility	*The data are accessible within this article. They are additionally publicly accessible on Mendeley* (https://doi.org/10.17632/rcgjrvdj8w.1). https://doi.org/10.17632/rcgjrvdj8w

**Table:** 

**Value of the data** • The data could be used for teaching in disciplinary literacy involving a range of specific-genres of writing such as abstracts, introductions, literature review, methods, findings, discussion, implications and conclusion. • It could be used for classroom interactive activities, formative assessment and feedback in disciplinary writing acquisition • The data has Neuro Linguistic Programming (NLP) import, as it can enable us to understand how L2 novice writing can develop from given background characteristics to approximate disciplinary community writing ethos. • It provides more detail on the role of language learning frameworks in the construction and communication of knowledge in the disciplines • The data can form a viable corpus for the analysis of different stages of disciplinary writing development

## 1 Data

The datasets in this article comprise information on the nature and role of systemic functional language features in L2 PhD students’ acquisition of writing within the disciplines at the university level [1]. In addition, the data depicts evidences of discourse-structural and linguistic feature changes due to practice inside the university classroom, which is based on interactive pedagogy [2], [3].

**Fig. 1:**
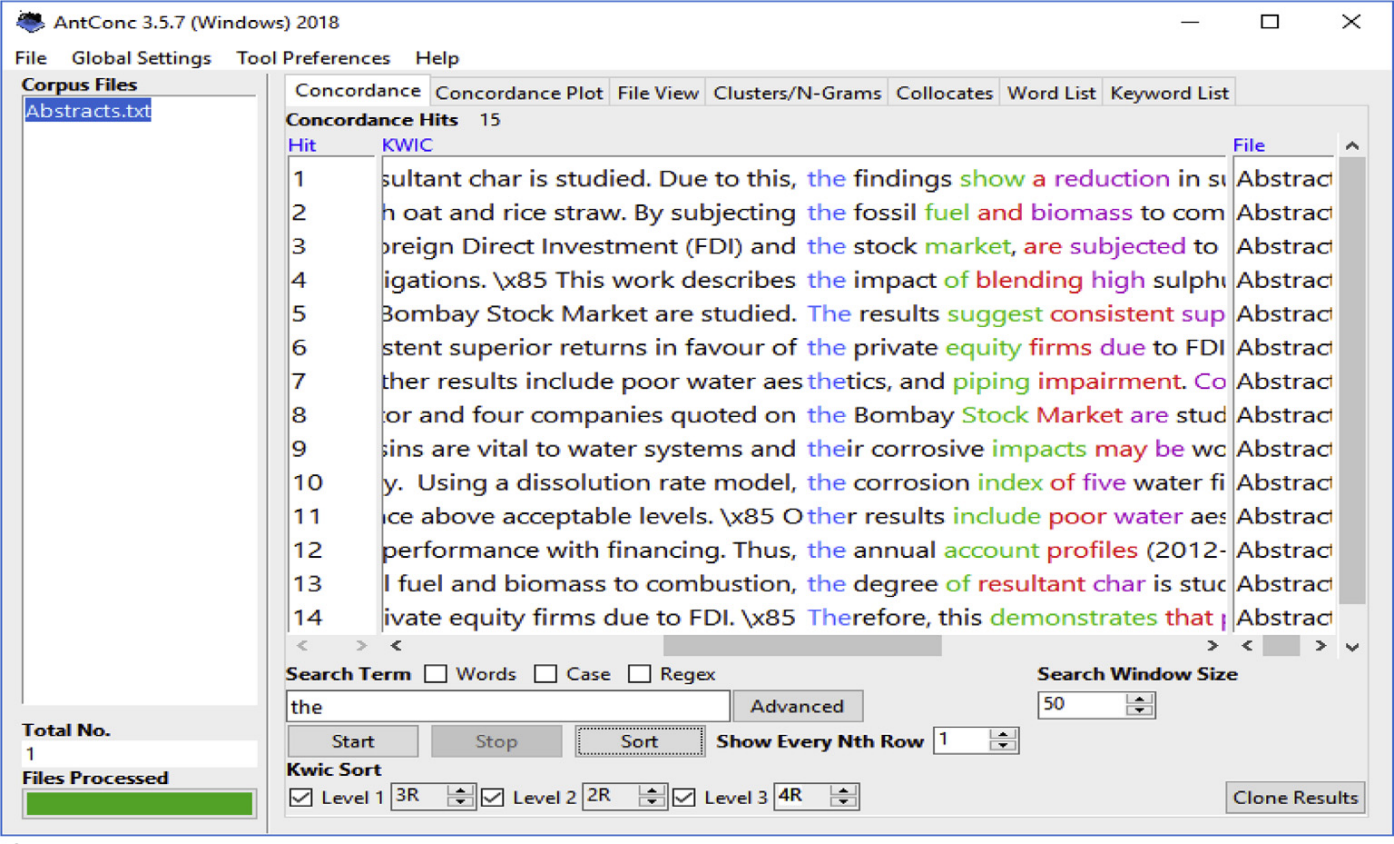
Concordance search to locate “findings/results” move in abstracts.

**Fig. 2:**
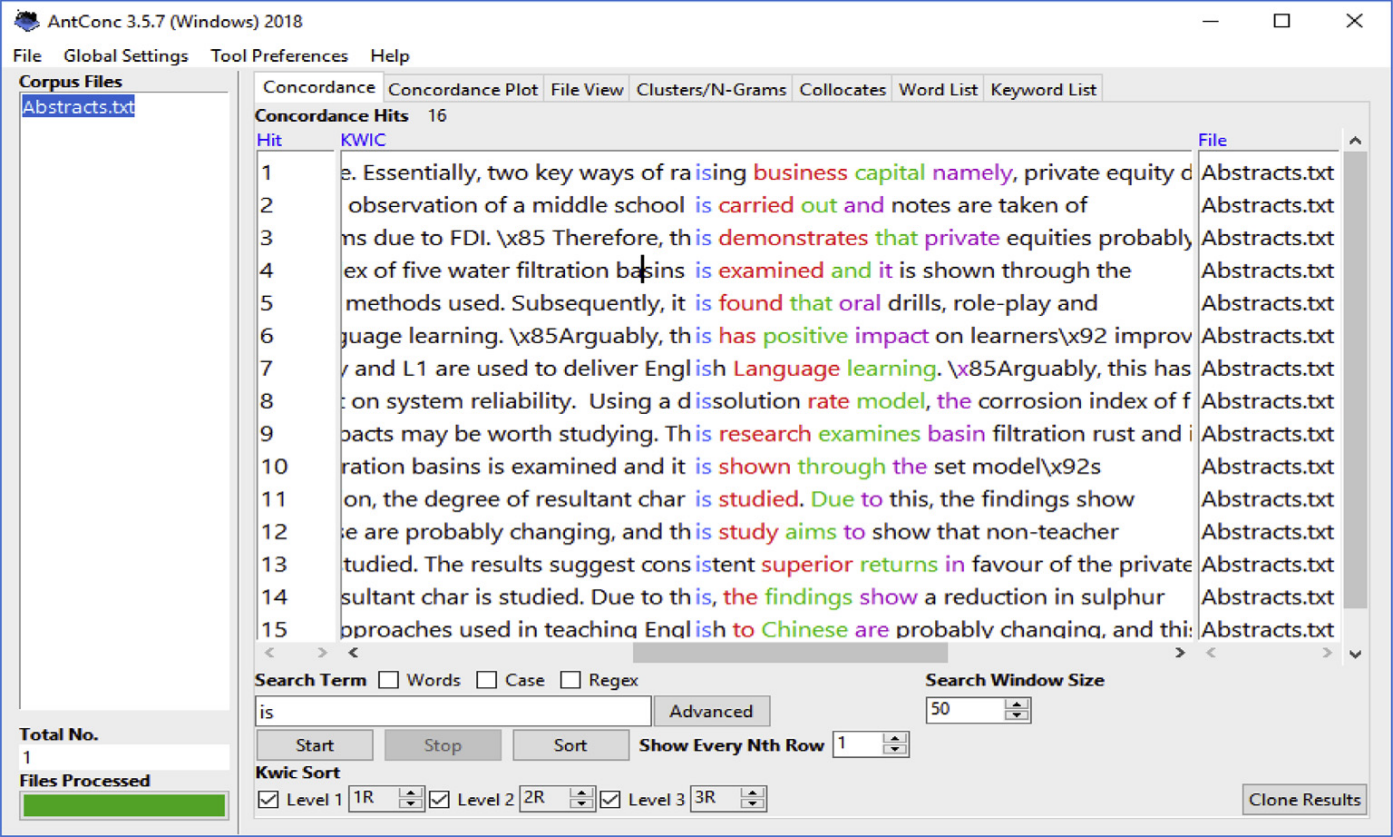
Concordance search to locate passive voice in the abstracts.

The datasets are presented in two parts. The first consists of three subsets, namely written conference abstracts (Table 1, Table 2, Table 3), raw marks for the assessed written abstracts which are further shown in paired t test results to evidence performance changes (Table 4, Table 5, Table 6, Table 7, Table 8), and AntConc software concordance search hits of the written abstracts’ discourse moves and linguistic features (Fig. 1, Fig. 2). The second dataset consists of qualitative questionnaire responses from the students about their experiences of the classroom intervention (Table 9).

**Table 1:** Science and Engineering participants’ unprocessed pre-and post-lesson written abstracts.

Pre-lesson conference abstracts	Post-lesson conference abstracts
**The impact of new architectural typologies on the lifestyle of the elderly in China** (Student 2, Sc & Engr, CHINESE) The aim of this paper is to study the changing housing architectural types in China and impact on the elderly lifestyle. The study has been out to research changes in the new structure building and effect on elderly social lifestyle. There have been 50 cases in a Chinese second-tier city considered, as far as the changes are more drastic for such second-tier cities in which the architectural changes are newer and fast developing. The second-tier cities witnessing such faster changes. The research found real differences which impact the life of the elderly in such aspects as access, space for relaxation, and space for exercising as healthy life.	Since China's opening-up policy in the 1980s, new architectural typologies have emerged different from traditional Chinese living houses. The housing architectural changes are fast spreading in China with effects on people's existence. This research studies the new architectural typologies impact on the lifestyle of the elderly, considered in this research to be aged 65 years and above. Comparative framework is used to investigates 50 cases of elderly residents in a Chinese second-tier city. Findings may show that compared to previous experiences in the traditional Chinese houses, the elderly now living in the modern architectural houses faces changes in access, relaxation spaces and natural sites for physical exercises.
**Water desalination through low-temperature energy sources** (Student 4, Sc &Engr CHINESE) Water centrality to human being for drinking and cooking and for other survival activities such as farming have stayed paramount. But over 70% of the world water lies in the ocean meaning that this amount of water is salty. Desalination become a vital process in reaching this vast amount of water. This study evaluate the technical advantages of key ways of water desalination with a view to suggesting an integrated cost-effective approach. Key ways of water desalination have included thermal, electrical and pressure. A combination of options needed to save cost in the process of desalination. This lead to effectiveness and higher water yield	Water is central to humans for direct consumption and for other activities such as farming. More than 70% of the world's water is sea water which is salty. This study evaluates the technical efficiency of main ways of water desalination including thermal, electrical and pressure. The approach is dependent upon the criterion of low-temperature energy sources which can lead to efficient distillation water desalination. Based on a laboratory-scale low temperature energy source from solar or waste heat, desalination can be effectively achieved at lower cost. An integrated cost-effective approach which depends on solar and waste energy is proposed for desalination at low temperatures.

**Table 2:** Business School (Buss) participants’ unprocessed pre-and post-lesson written abstracts.

Pre-lesson conference abstracts	Post-lesson conference abstracts
**The Belt and road initiative as a tool for peace in South-East Asia** (Student 6, Buss, CHINESE). Since 2008 and beyond when China's rise became obvious to the world due to the economic downturn witnessed in the developed world, tensions have also become noticed in the media between China and countries around it, the geographically and demographically smaller nations. Such tensions mostly centred around claims of small islands ownership between China on the one hand and each of the individual smaller countries. By 2013 the Chinese President, Xi Jinpin proposed a belt and road initiative, it simple means providing infrastructure based on transportation networks to link China with all parts of the world for economic development. This study argue that the successful belt and road project can bring about peace and stability, in particular south-east Asia region. We will show that a better transportation and communication link the south-east Asia nations and China will lead to people economic link and the prosperity of the region which will reduce the feeling of inequalities that encourage tension.	Since 2008 and beyond when China's rise became obvious to the world due probably to the economic downturn witnessed in the developed world, tensions have also become noticeable in the media between China and countries around it which are geographically and demographically smaller. This study argues that the belt and road project. Proposed by the Chinese President Xi Jinpin in 2013, can bring about peace and stability, in south-east Asia. The argument is anchored on the notion that a better transportation and communication link between the south-east Asia nations and China will lead to more people to people economic link as well as the prosperity of the region. Consequently, the prosperity will reduce the feeling of economic inequalities that often encourage tensions. Currently, such tensions seem worsened by an image of Chinese hegemony painted to the smaller countries in the region by external powers such as the United States. By implication, a prosperous south-east Asia with strong middle class society will yield less to military war as a solution to the contentions.
**Innovation as a catalyst for firm's change and sustenance in the Shanghai area of China** (Student 7 Buss, CHINESE) There are current approaches to the understanding of companies' growth with the role played by innovation stressed. The stress is on technological innovation as separately connecting to internal strengths, regional status and social systems, independently treated. A systemic approach is however seen to be better to gauging the performance of firms in that a holistic picture perhaps developes for efficiency in planning and innovation. This research therefore aims to eclectically study the innovations which businesses are targeting by examining internal, regional and social factors together, instead looking at them separately. The emphasis is on examining the logistic operations of the selected companies within China, and the results are shown to have likely extents of innovation successes	Innovation is increasingly seen as a core measure for the assessment of companies' growth, although it is often separately linked to internal strengths, regional status and social systems. This research aims to eclectically investigate the innovative status of selected companies in the Shanghai area of China by eclectically examining their internal, regional and social links factors together in relation to innovation. This approach is can paint a holistic picture of the innovative efficiency of the companies. Therefore, empirical cases of the selected companies are examined with emphasis on their logistics systems. The possible findings indicate a mixed picture of a strong shift towards digital-based innovative systems and a residue of the traditional labour-intensive structures. Thus, some understanding can be gained as regards the direction of change in the next two decades to be experienced by companies that aim to remain competitive and thriving.

**Table 3:** Humanities and Social Science participants’ unprocessed pre-and post-lesson written abstracts.

Pre-lesson conference abstracts	Post lesson conference abstracts
**Cultural representations in digital translations: A study of WeChat** (Student 8, Hum & Soc Sc, JAPANESE) This research has aimed to show that the spread of IPhone worldwide has come with the innovative idea of applications for ease of communication and other commercial functions. Along with languages of communication on the apps such as English and Chinese, translation modes have been further encrypted allowing then for ease of communication. In this article, we will examine the Chinese to English, and vice versa, translations on WeChat app. The aim has been to find out the cultural representations which exist in such translations. The notion of cultural representation and thought process as held and conveyed through language will be used by this research to examine the patterns of Chinese to English translations on WeChat.	The spread of IPhone worldwide has come with the innovative idea of applications for ease of communication and other commercial functions. To make communication across language boundaries even easier, translation modes have been encrypted on the apps. In this article, we intend to deal with research that examines Chinese to English, and vice versa, text translation on WeChat app in order to explore the cultural representations which can be found in such translations. The framework of cultural representation, which also concerns how thought process is held and conveyed in language, is used to study patterns of Chinese to English and English to Chinese translations on WeChat. Results are likely to show meaning convergences as well as total deviations from source to target languages. This calls for greater interaction between translation software specialists, linguists and professional translators to strengthen bilingual communication on platforms such as WeChat
**Misused and omitted prepositions in the written English of Chinese first year university students** (Student 10, Hum & Soc Sc, CHINESE) Conceptual expressions in the indication of relationships between nouns and verbs through prepositional use differ between English and Chinese languages. In their written English, Chinese learners of English can vary the use of prepositions as influenced by notions in Chinese thought and communication. Effectively, this study examines cases when Chinese first-year university students fail to use or misuse prepositions in their written English. The framework of conceptual transfer common in second language acquisition is used by this research to show the cases of omission and misuse of prepositions by the students. The findings show that meaning misrepresentation occur in the written English of the Chinese first-year university students, which can affect clear meaning and expressions of the students.	Conceptual transfer from L1 to L2 possibly affect the expression of meaning in various ways, including stating the relationship between nouns and verbs through the use of prepositions. In effect, this this study carries out a corpus analysis of the written English of Chinese first-year university students to examine cases when the students fail to use or misuse prepositions due to conceptual transfer from their L1. The conceptual transfer framework is used to show how L1 affects L2 in the usage of prepositions. Word-chunks are isolated and these are analysed to show the relationship among the words as far as prepositional consequences go. The results demonstrate that prepositional omissions and misuse in the students' written English are perhaps based on conceptual transfers from L1 which lead to deviations in L2 prepositional use. Similarly, some of the L2 deviations impede communication while others do not. Therefore, while efforts can be made to remedy some usages of prepositions by Chinese learners, other manifestations might be recognised as deviations which do not disturb communication.

**Table 4:** Pre- and post-interventions raw scores on a scale of 1–5.

Student	Pre-Lesson/Post-Lesson Scores	Inclusion of moves					Occurrence of linguistic features			
		M1	M2	M3	M4	M5	PS	PASV	HEDG	COH
Student 1 JAPANESE (Sc & Engr)	PreL Score	1	3	3	2	1	2	2	1	2
	PstL Score	4	5	5	5	4	4	4	4	3
Student 2 CHINESE (Sc &Engr)	PreL Score	2	3	2	2	1	1	2	1	2
	PstL Score	4	4	5	5	4	4	4	3	3
Student 3 NIGERIAN (Sc &Engr)	PreL Score	2	3	2	2	2	2	2	2	1
	PstL Score	5	5	5	5	4	5	4	3	4
Student 4 CHINESE (Sc &Engr)	PreL Score	2	2	3	2	1	2	2	1	2
	PstL Score	4	5	5	5	4	4	4	3	3
Student 5 INDIAN (Buss)	PreL Score	2	3	3	3	2	2	3	3	2
	PstL Score	5	5	5	5	4	5	5	5	5
Student 6 CHINESE (Buss)	PreL Score	3	4	4	3	2	2	3	3	2
	PstL Score	5	5	5	5	4	5	5	4	4
Student 7 CHINESE (Buss)	PreL Score	3	3	3	3	2	3	4	3	2
	PstL Score	4	5	5	5	4	5	5	4	4
Student 8 JAPANESE (Hum & Soc Sc)	PreL Score	1	3	3	3	2	2	2	3	2
	PstL Score	5	5	5	5	4	5	4	5	4
Student 9 CHINES (Hum & Soc Sc)	PreL Score	2	3	2	3	2	2	3	2	2
	PstL Score	5	5	5	5	4	5	5	5	5
Student 10 CHINESE (Hum & Soc Sc)	PreL Score	2	2	2	3	2	2	2	2	2
	PstL Score	4	5	5	4	4	4	4	4	5

**Table 5:** Paired t-test of CA moves inclusion in the pre-and post-lessons.

t-test	Pre-Lesson test	Post-Lesson test
Mean	25673.4	52118.9
Variance	45893734.0444	20978332.1
Stand. Dev.	6774.4914	4580.2109
Number	10	10
t-test 15.2204		
degrees of freedom 9		
critical value 2.262		

The absolute value of the calculated t exceeds the critical value (15.2204 > 2.262), so the means are significantly different at p < 0.05.

**Table 6:** Paired t-test of CA linguistic features (PS, PASV, HEDG, and COH) occurrence.

t-test	Pre-Lesson test	Post-Lesson test
Mean	2272.9	5084
Variance	276976.9889	310342.8889
Stand. Dev.	526.286	557.0843
Number	10	10
t-test 16.7792		
degrees of freedom 9		
critical value 2.262		

The absolute value of the calculated t exceeds the critical value (16.7792 > 2.262), so the means are significantly different at p < 0.05.

**Table 7:** Comparison of HD and SD Post-lesson moves inclusion.

t-test	Post-lesson HD	Post-Lesson SD
Mean	4.6	4.7
Variance	0.2526	0.2172
Stand. Dev.	0.5026	0.466
Number	20	30
t-test 0.7092		
degrees of freedom 39		
critical value 2.023		

The absolute value of the calculated t is smaller than critical value (0.7092 < 2.023), so the means are not significantly different.

**Table 8:** Comparison of HD and SD Post-lesson linguistic features inclusion.

t-test	Post-lesson HD	Post-Lesson SD
Mean	3.6875	4.625
Variance	0.3625	0.2172
Stand. Dev.	0.6021	0.4946
Number	16	24
t-test 5.1738		
degrees of freedom 28		
critical value 2.048		

The absolute value of the calculated t exceeds the critical value (5.1738 > 2.048), so the means are significantly different.

**Table 9:** Students’ experiences of the classroom intervention.

Participants	Learning experience
Student 10	The work about understanding the other abstracts given to us when we were in the lesson made me feel like I was learning from other experienced writers to do my own correction and writing of the conference abstract in my field.
Student 3	I compared the abstract I studied in my area with students from other subjects and I feel there is some difference between engineering abstracts and abstracts in arts faculty. Engineering abstracts are shorter and they go straight to the point. I am learning to write like this.
Student 6	Splitting the abstracts into components made it easier for us to understand the content structures. Knowing that the abstract has key moves became easier. I used this to correct my draft
Student 8	I was able to find out myself the structure parts within an abstract now I know that I can write an abstract in five movements. Before I tried to write an abstract like a summary of the paper I am working on
	The exercises [activities] helped us to know what should be included in an abstract
Student 6	My attention was drawn to language patterns that I can now write properly in abstracts. I can state ideas in a more indirect style that is more academic and can link the ideas to flow on nicely
Student 10	The conference call for papers helped me to find a topic. Working together on it in class made me to read it more carefully and the breakdown of themes made a lot of sense to me I build a topic from one of the themes.
Student 5	Lively and chatty. I expected that the teacher will tell us everything about writing conference abstracts like in a normal university lecture but we did all the talking and discussion. I think that I was able to learn more like this way by practice with others.
Student 4	Like a workshop we found out things ourselves by exchanging ideas and breaking down many sentences in the abstracts we were given in the class. I copied this to improve my old abstract draft.
Student 1	Exchanging arguments with PhD students from the arts and social science make the learning very meaningful to me. I am now appreciative of how abstracts writing in my field is different from other fields.
Student 7	For me I think the different stages during the lesson was a step by step way of learning how to write and correct abstracts. It is like the skill of writing, checking, comparing and changing sentences to write ideas that will be acceptable for conference presentation.
Student 2	As a PhD student in Engineering, it is a common practice to work in groups. Hence this aspect of the lecture was very convenient. It helped me to correct my draft abstract

### 1.1 Written and marked conference abstracts dataset

The first dataset is based on conference abstracts (CAs, henceforth) written by ten focal L2 PhD students at a Sino-British university who participated in a classroom instruction aimed at improving their disciplinary literacy. They were worked with on an English for Research and Publication course. The first subset of the written CA data is a combination of unprocessed pre- and post-classroom intervention CAs (Table 1, Table 2, Table 3).

Table 1 contains pre- and post-lesson written CAs by each of the two science and engineering participants. Differences can be seen between the pairs of abstracts (pre-versus post-lesson), which reflect a comparatively superior writing ability after the lesson intervention. Table 2 contains similar CA pairs written by participants from the business school.

Table 2 shows that knowledge in the business discipline is also better constructed after the classroom practice. The post-instruction CAs can be more clearly followed than those written before the intervention. The combined pre- and post-classroom intervention CAs in the humanities and social sciences are presented on Table 3.

The CA writing improvement after the classroom intervention, as witnessed in the output of the science and engineering as well as by the business school participants, is also replicated by the humanities and social science students on Table 3.

The second subset, within the written CA dataset are scores which were awarded to both the pre- and post-lesson CAs of the ten focal students. The raw scores for the ten focal students are shown on Table 4. To measure the significance of the difference (over time and acquisition improvement) between the pre- and post-intervention CA scores that are found on Table 4, paired t test results involving the ten focal students are presented on Table 5, Table 6, Table 7, Table 8

In relation to the performance changes, the paired t test data are shown for: CA structure-moves (Table 5); CA linguistic features (Table 6); variation between what Hyland [4] refers to as the Hard Disciplines (HD-science and engineering) and the Soft Disciplines (SD-humanities and social sciences) in CA structure-moves (Table 7); and in linguistic features (Table 8).

A third data subset, from the written and marked CA dataset, which comprises concordance search results, is presented on Fig. 1, Fig. 2. Only the focal students' post-intervention CAs were selected and then processed for concordance hits. Fig. 1, Fig. 2 respectively contain data showing the hits for a search to locate the ‘findings/results’ discourse-move, and for another search to detect the linguistic feature of ‘passive’ voice.

### 1.2 Learners’ experiences of the classroom intervention

The second dataset is made up of the focal students’ empirical experiences of the classroom intervention. The views are presented in Table 9.

## 2 Experimental design, materials, and methods

To acquire the above datasets, twenty-three PhD students at the Sino-British university took part in the intervention lesson on CA writing. However, only the data related to 10 focal students are collected and presented in this article. Before the process of requesting for any written CAs and delivering the lesson, ethics approval was sought and obtained from the research ethics committee of the Sino-British university. The focal participants were also clearly informed about the research and they provided informed consent for the use, under anonymity conditions, of information gathered from them for research purposes.

### 2.1 Collection of written and marked CA dataset

Prior to the lesson, the students were each asked to write an abstract on a chosen topic in their discipline, and were supported with respective disciplinary conference calls for abstracts. The pre-lesson CAs were based on topics connected to the conference calls. During the lesson, the students worked to improve the pre-lesson CAs which they had drafted. After the lesson, ten focal students were then selected using stratified random sampling. They were worked with over a one month period via the university Moodle and emailing platforms to further improve the discourse-moves and linguistic features of their CAs. The pre- and post-lesson CA data presented in this data article are those connected to only the ten focal students.

Moving on to the scores data, pre- and post-intervention CAs written by the ten focal students were subjected to a grading process using a 1–5 scoring descriptor instrument (see Table 10) which was adapted from the Sino-British university's academic writing assessment rubric. The grades were awarded after a triple-blind anonymised marking of the CAs. Paired t test was then used to compare the significance of the pre-and post-intervention CA scores.

**Table 10:** Marking descriptors.

Criteria	Possible Mark	Descriptors	Earned Mark
Inclusion of CA structure moves	5	-“Core” moves inclusion (e.g. Move 2: research aim/problem; Move 3: method/procedure used; Move 4: findings). -Abstract is in single paragraph, key words are listed at the bottom. -Some of these are present: identification of research gap, contribution to knowledge, theoretical framework.	
	4	-“Core” moves included. -Abstract in single paragraph, and lists key words at the bottom. -Research problem is included	
	3	-Majority of moves are included with one “core” moves missing. -Abstract is in single paragraph. Key words are listed below it.	
	2	-Majority of moves included with two “core” moves missing. -Abstract is in single paragraph. Key words are not listed.	
	1	-Only one or no “core” move is included. -Abstract is written in two or more paragraphs and there are no key words listed.	
Use of relevant tenses	5	-Present simple/present perfect/past simple used to express the research “need issue” or gap/problem, research aim (e.g. in moves 1&2). -Past simple is used to state how the data was collected (e.g. in Move 3). Present simple, past simple/present perfect used to state how the analysis was done. -Results are stated, e.g. in the present simple, past simple. -Implications expressed, e.g. in present simple, present perfect.	
	4	-Present simple, past simple and present perfect are mostly appropriately used. There are very few problems of verb agreement and verb tense.	
	3	-Present simple/past simple over-used and incorrectly mixed with other tenses. -There are problems with verb agreement. No verb tense which interfere with clarity of communication.	
	2	-Present simple, past simple are hardly used. There are significant problems of verb agreement and verb tense, which make meaning unclear/place a strain on the reader.	
	1	-Majority of the verbs are incorrectly used -Communication significantly breaks down and the reader is highly strained.	
Inclusion of hedging	5	-Hedging is correctly used, e.g. to state research gap (Move 1) and implications (Move 5). -Hedging is skillfully and appropriately used in other move structures.	
	4	-Hedging is used, e.g. in either Move 1 or Move 2, and/or is correctly attempted in other moves.	
	3	-Hedging is not used in Move 1 or 2. -May be correctly attempted in other moves.	
	2	-Hedging is incorrectly attempted in all moves	
	1	-Hedging is not attempted anywhere in the abstract.	
Use of passive	5	-Passive is used, e.g. in methodology (Move 3), findings (Move 4) (e.g. with past simple/present perfect). -Passive is highly appropriately used in other moves	
	4	-Passive is used in either Move 3 or Move 4. -It is attempted in other moves	
	3	-Passive in neither used in Move 3 nor 4 but is attempted in other moves.	
	2	-Passive is nether used in Move 3 nor 4 and is unsuccessfully attempted in other moves	
	1	-Passive not attempted anywhere in the abstract	
Inclusion of cohesive markers	5	-Sophisticated argument and line of logic -Consistent and appropriate use of cohesive markers.	
	4	-Some level of sophistication in argument with a clear logic -Appropriate use of cohesive markers with minor inconsistencies	
	3	-Clear argument with occasional breakdowns -Cohesive devices are adequately uses but there are minor inconsistencies	
	2	-Argument and line of logic can be followed but with frequent breakdowns -Acceptable use of cohesive devices but there are inconsistencies	
	1	- Argument and line of logic can be discerned but requires reader's effort -Cohesive markers are mostly inappropriately used	

Regarding the concordance data, only the post-intervention CAs of the focal students were uploaded on AntConc (Windows 3.5.7), a software application for corpus analysis. Concordance searches were then carried out to check the extent of the intervention's impact on the focal students' post-intervention CAs in terms of ability to include structure-moves and linguistic features.

### 2.2 Gathering of participants’ learning-experience dataset

The second dataset was collected by means of qualitative questionnaire prompts (Table 11) which allowed the participants to freely share their experiences of the intervention. Care was taken to ensure that the prompts were clear, specific, but without leads and bias.

**Table 11:** Qualitative Data Collection: Semi-structured questions for the collection of learner's classroom experiences Instruction: Looking back at the activities and interactions in the class on conference abstract writing, describe how you feel/felt. For each of the below listed classroom practice aspect, freely state your experience^a^.

Question no	Question-prompt as focused on classroom practice aspect
1	Sample abstracts used in class during the lesson
2	Example linguistic features used during the lesson
3	Worksheets used during the lesson (specify where you possibly can)
4	Conference calls for abstracts used in class
5	Other materials you would wish to mention
6	The classroom activity on “understanding the entire abstract components”
7	The classroom activity on “understanding the units within an abstract paragraph”
8	The class activities on re-drafting your abstracts
9	Working with fellow students within your discipline during the class pair and group activities (please, provide specific examples)
10	Working with students outside of your discipline during the class pair and group activities (please, refer to specific examples)
11	After each pair or group work which you can recall, what do you think about the way in which the pair/group reports to the entire class were presented?
12	Any other aspect of the classroom lesson not mentioned above that you want to comment on

a Ample spacing was provided for the free writing of comments in the originally administered questionnaire.

### 2.3 Classroom intervention activities

**Fig. 3:**
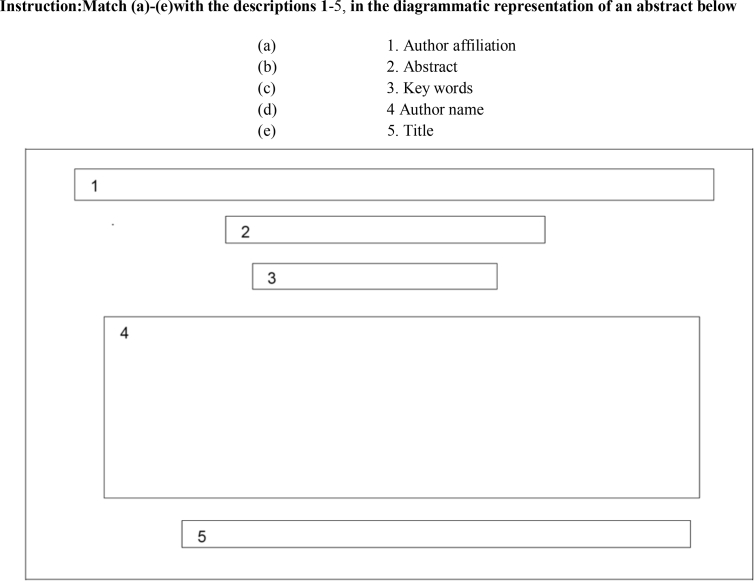
Classroom activity 1: Identification of abstracts overall components.

Given that classroom practice research in disciplinary literacy is evolving [3], it is vital to share classroom intervention activities which can further elucidate the post-intervention CA data presented in this article. For example, the intervention activities in Fig. 3, and Table 12, Table 13 (particularly Table 12, Table 13), contributed to contextualizing the focal students’ acquisition abilities, which enabled them to produce the post-intervention CA data.

**Table 12:** Classroom activity 2: Analysis, awareness and acquisition [6] of abstract-moves.

Activity	Instruction: Using the provided model abstract in your discipline, complete the below activities:
(a)	Work alone, find and underline the following word-chunks or other words like them:
	(i) “current practices show that”, “… calls attention to …”, “numerous studies have been done in this field but there are significant gaps in”, etc. (e.g. Jiang & Hyland [5])
	(ii) “this study aims to … /seeks to … /explores/explored/the aim of this study was”, etc. (e.g. Jiang and Hyland [5])
	(iii) “the gap is addressed by exploring …”, “using a method known as …”, “by subjecting … to …”, etc. (e.g. Swales and Feak [6])
	(iv) “the findings suggest”, the results show”, “the finding demonstrate”, etc. (e.g. Hyland [4])
	(v) “these data show that”, “our findings clearly demonstrate that”, these results suggest that”, “arguably, this means that”, etc. (e.g. Swales & Feak [6])
(b)	Compare your results with someone in your discipline, and discuss the idea expressed to the right-hand side of your underlined word-chunks
(c)	Work alone and match the following “move-structures” (Hyland [4]) to the word-chunk categories in activity (a):
	(i) Move 2: Purpose/Aim, (ii) Move 5: Conclusion/Implication, (iii) Move 3: Method, (iv) Move 1: Introduction, (iv) Move 4: Product/Result
(d)	Compare your practice in activity (c) with another person's practice. That person should be from a different discipline category (Hard Discipline versus Soft Disciplines):
	-What similarities and differences do you find in the types of moves used in your discipline and in the other person's specialist area?

**Table 13:** Activity 3: Analysis, awareness and acquisition (Swales & Feak [6]) of abstracts linguistic features.

Activity	Instruction: Read the provided model abstract in your field and answer the questions below:
(a)	Individually read the model abstract and underline these language features: (i) Present simple, (ii) Cautious language (hedging), (iii) Passive voice (iv) Specialist keywords, (v) Cohesive links
(b)	Compare your practice in (a) with another person's practice. That person should be from a different discipline category (Hard Discipline versus Soft Disciplines): What similarities and differences (between your disciplinary abstract and the other person's) do you notice in the occurrence of the linguistic feature which you underlined in (a). Clues: Are there more, less, similar usages of cautious language, passive, cohesive links? What type of words (e.g. simple/monosyllabic, complex/multisyllabic, modal) are frequently used as cohesive links? Are strings of nouns frequently used to express a concept?
