# Supplementary material for: Datasets evidencing research on classroom practice in L2 disciplinary writing
Source: Data Brief. 2019 Jul 9;25:104222. doi: 10.1016/j.dib.2019.104222 (PMC6661450; doi:10.1016/j.dib.2019.104222)
Supplement: Supplementary file 1 [file mmc1.zip › EXCEL FILES and Word File as Supplementary Materials/FILE #9-11 Figures (1), (2), (3) .docx]

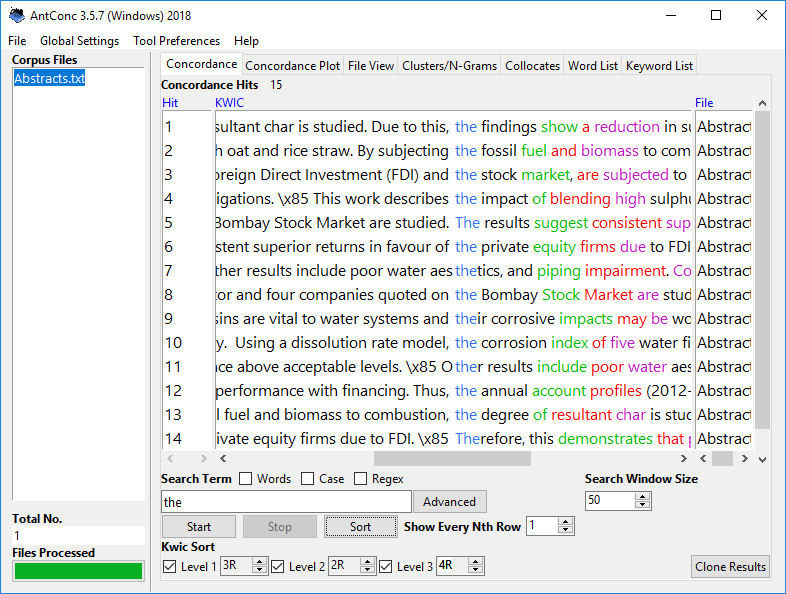


**Figure 1**

**Concordance search to locate “findings/results” *move* in abstracts**


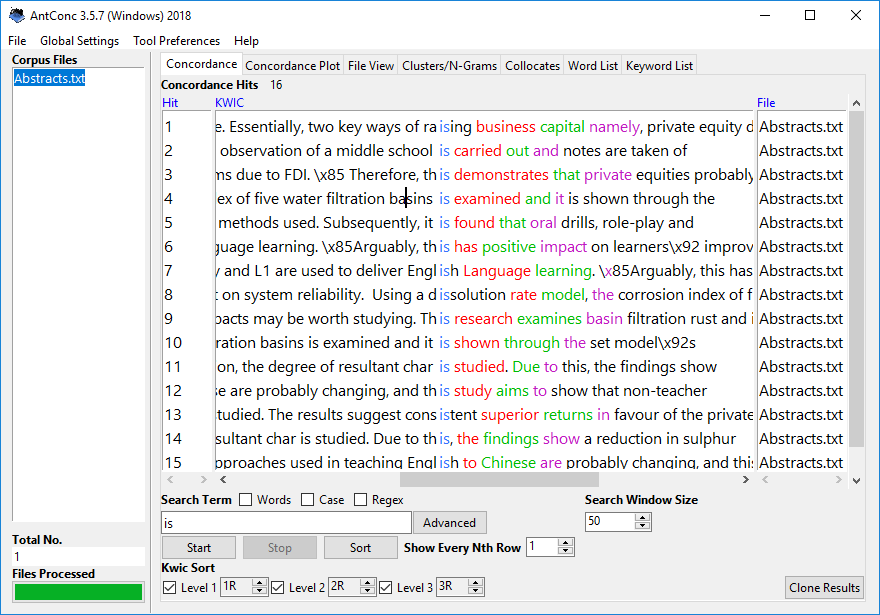


**Figure 2**

**Concordance search to locate passive voice in the abstracts**

**
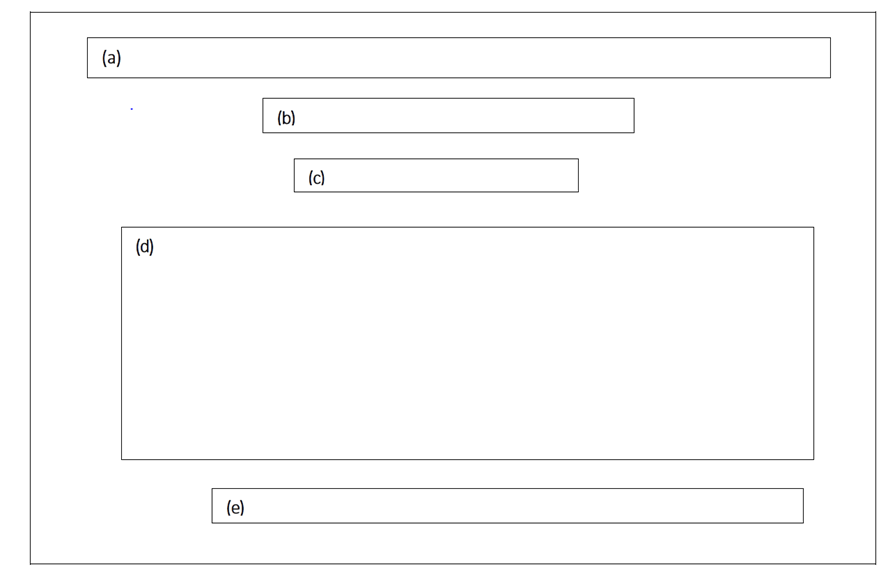
**

**Figure 3**

Classroom activity 1: Identification of abstracts overall components
